# Strong differences in the clonal variation of two *Daphnia *species from mountain lakes affected by overwintering strategy

**DOI:** 10.1186/1471-2148-11-231

**Published:** 2011-08-08

**Authors:** Eva Hamrová, Joachim Mergeay, Adam Petrusek

**Affiliations:** 1 Department of Ecology, Faculty of Science, Charles University in Prague, Viničná 7, CZ-12844 Prague 2, Czech Republic; 2 Laboratory of Aquatic Ecology and Evolutionary Biology, University of Leuven, Deberiotstraat 32, B-3000 Leuven, Belgium; 3 Research Institute for Nature and Forest, Gaverstraat 4, B-9500 Geraardsbergen, Belgium

## Abstract

**Background:**

The population structure of cyclical parthenogens such as water fleas is strongly influenced by the frequency of alternations between sexual and asexual (parthenogenetic) reproduction, which may differ among populations and species. We studied genetic variation within six populations of two closely related species of water fleas of the genus *Daphnia *(Crustacea, Cladocera). *D. galeata *and *D. longispina *both occur in lakes in the Tatra Mountains (Central Europe), but their populations show distinct life history strategies in that region. In three studied lakes inhabited by *D. galeata*, daphnids overwinter under the ice as adult females. In contrast, in lakes inhabited by *D. longispina*, populations apparently disappear from the water column and overwinter as dormant eggs in lake sediments. We investigated to what extent these different strategies lead to differences in the clonal composition of late summer populations.

**Results:**

Analysis of genetic variation at nine microsatellite loci revealed that clonal richness (expressed as the proportion of different multilocus genotypes, MLGs, in the whole analysed sample) consistently differed between the two studied species. In the three *D. longispina *populations, very high clonal richness was found (MLG/N ranging from 0.97 to 1.00), whereas in *D. galeata *it was much lower (0.05 to 0.50). The dominant MLGs in all *D. galeata *populations were heterozygous at five or more loci, suggesting that such individuals all represented the same clonal lineages rather than insufficiently resolved groups of different clones.

**Conclusions:**

The low clonal diversities and significant deviations from Hardy-Weinberg equilibrium in *D. galeata *populations were likely a consequence of strong clonal erosion over extended periods of time (several years or even decades) and the limited influence of sexual reproduction. Our data reveal that populations of closely related *Daphnia *species living in relatively similar habitats (permanent, oligotrophic mountain lakes) within the same region may show strikingly different genetic structures, which most likely depend on their reproductive strategy during unfavourable periods. We assume that similar impacts of life history on population structures are also relevant for other cyclical parthenogen groups. In extreme cases, prolonged clonal erosion may result in the dominance of a single clone within a population, which might limit its microevolutionary potential if selection pressures suddenly change.

## Background

Many organisms are capable of alternating sexual and asexual reproduction throughout their life cycle, including protists, cnidarians, rotifers, crustaceans, insects, mosses, vascular plants and macro-algae [1]. The relative importance of both reproduction strategies has profound influences on the genetic structure of populations and on the evolutionary response to selection pressures [2]. In many cases, sexual reproduction in such taxa is associated with the formation of dormant stages that ensure population survival during unfavourable periods (i.e., dispersal through time), as well as spatial dispersal [3].

In aquatic environments that undergo regular seasonal changes, including cold temperate lakes or temporary ponds, many zooplankton groups such as water fleas (Cladocera) usually survive unfavourable periods of winter or drought as dormant stages, and hatch at the onset of more favourable conditions. The typical cladoceran life cycle (reviewed in [2]) is thus cyclical parthenogenesis: females reproduce clonally under favourable conditions, and switch to male production and sexual reproduction when conditions deteriorate.

Fertilized dormant eggs, usually encased in an ephippium, enter dormancy at an early embryonic stage, endure unfavourable periods, and can hatch when the conditions improve again. Each hatchling from a dormant egg of a cyclically parthenogenetic cladoceran has a unique genotype [4,5]. Under this scenario, a high number of genotypes may be expected at the beginning of the growing season in such populations. However, due to selection and drift during the prolonged period of parthenogenetic reproduction, some clonal lineages disappear, resulting in clonal erosion and a reduction in effective population size. Apart from perceivable reductions in clonal and genetic diversity, this typically leads to deviations from the Hardy-Weinberg equilibrium, and increases among-population genetic differentiation as a result of enhanced genetic drift [4-6].

During parthenogenetic reproduction in the growing season, genotypes best adapted to their local environment are favoured. This process often results in the coexistence of a limited number of genotypes [7,8]. Clonal diversity is restored by sexual reproduction and the subsequent hatching of recombinant genotypes. Additionally, genetic diversity can increase through hatching of older dormant eggs produced during earlier growing seasons [9]. However, prolonged periods of clonal selection, though benefiting certain clones, may have a negative impact on the population as a whole. Populations where sexual reproduction is infrequent have lower effective population sizes, and sometimes suffer from inbreeding depression [10].

The above-mentioned processes are of course not limited to cladocerans. The genetic structure of cyclical parthenogen populations in general is affected by the strength of the clonal selection, length of the growing season, and frequency of sexual reproduction [1,5]. A reduction of clonal diversity over time has also been observed in natural populations of rotifers (e.g., [11]), aphids (e.g., [12]), as well as plants (e.g., [13]). Similarly, the structure of strictly asexual populations composed of different clones is affected by the frequency of formation or immigration of new clones, and clonal decay [14]. If new clones are not replaced with sufficient frequency, a reduction of clonal diversity over time can be expected due to neutral processes, even in the absence of any selection advantage of particular clones [15].

An important group in which these phenomena have been studied in detail is the cladoceran genus *Daphnia*. These small crustaceans are key grazers of phytoplankton in temperate lakes. In such habitats, *Daphnia *have two options to survive unfavourable winter conditions [16]: 1) as dormant eggs, re-colonizing the water column in spring as hatchlings; or 2) as active parthenogenetic females overwintering in the water column. These two strategies are not mutually exclusive, as a *Daphnia *female may produce dormant eggs sexually and subsequently switch back to parthenogenetic reproduction [17]. Variation in overwintering strategy has been observed both between *Daphnia *species inhabiting the same lake (e.g., [18]) and within the population of a single species (e.g., [16]). It has been also documented that there is a substantial impact of different recruitment strategies on the genetic structure of a *Daphnia *population among years with different weather conditions (in particular, warm vs. cold winters) [19]. In the case of a successful overwintering of maternal genotypes to the next growing season, the period of clonal selection may span many years, much longer than for populations relying on regular re-establishment from the dormant egg bank [4,5]. Individual-based modelling [6] predicts that the length of the parthenogenetic reproduction phase in the *Daphnia *life cycle may be crucial for the resulting clonal diversity. Therefore, different overwintering strategies are expected to have very different impacts on the clonal structure of *Daphnia *populations, their effective population size, and in the long term on their microevolutionary potential [2].

Accurate analyses of *Daphnia *clonal diversity, however, have long been hindered by a lack of suitable genetic markers. Most studies in the past have used allozyme electrophoresis with limited genetic resolution to detect multilocus genotypes (e.g., [8,19,20]). While this method has brought valuable insights into processes shaping populations diversity and structure, it is likely that the clonal variation in natural populations was often substantially underestimated [21]. The development of variable microsatellite markers for *Daphnia *species has allowed much more sensitive genetic analyses, and increased the chances that different clones are properly identified, and true clonal diversity better estimated (e.g., [16,21,22]).

**Figure 1:**
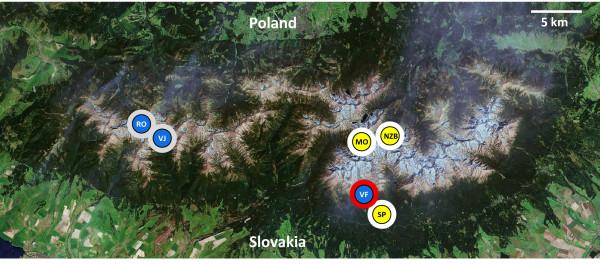
**Geographic location and the type of overwintering strategy of the sampled *Daphnia *populations (abbreviations as in Table 1)**. Species are differentiated by coloration of the inner circle: *D. galeata *populations are marked by black letters in yellow circles, *D. longispina *by white letters in blue circles. The outer ring indicates the overwintering strategy: white for active overwintering under the ice, grey for putative overwintering as dormant eggs only, red for confirmed overwintering as dormant eggs only. The satellite photo of the Tatra Mountain range is based on orthorectified Landsat 7 data (source: U.S. Geological Survey).

In this study, we focus on the variation in clonal structure of two *Daphnia *species inhabiting mountain lakes in relation to their presumed overwintering strategy. We studied six *Daphnia *populations from lakes in the Tatra Mountains (20°10'E, 49°10'N; the highest mountain range of the Carpathians, on the border between Slovakia and Poland; Figure 1) belonging to two closely related species of the *Daphnia longispina *complex, *D. galeata *and *D. longispina *(see [23] for phylogeny and nomenclatural issues in this complex). Both studied species are found in several mountain lakes of the region [24]. These taxa reproduce by cyclical parthenogenesis, and overwintering strategies for several local populations are known. In three lakes inhabited by *D. galeata*, individuals have been recorded under the ice, while in another locality inhabited by *D. longispina *no such individuals have been found (Table 1, Figure 1). This latter population presumably survives the winter in the form of dormant eggs only, although overwintering of a small number of females, with densities below the detection threshold, can never be completely excluded. We assume that similar strategies may occur in other populations living in environmentally similar conditions.

**Table 1:** Information about sampled localities and estimates of genetic diversity of the studied *Daphnia *populations

Lake	code	altitude (m)	**Z**_**max **_**(m)**	area (ha)	N	MLG	clonal richness	clonal diversity	polym. loci	alleles/locus	Fis	He	Ho	**HWE dev**.	overwintering
*D. longispina*															
Dolné (Prvé) Roháčské	RO	1562	7.7	2.2	37	37	1.00	1.00	9	4.6	0.01	0.49	0.49	no	N/A
Vyšné Furkotské	VF	1698	2.4	0.4	36	35	0.97	1.00	8	2.9	0.13	0.40	0.36	no	not observed [40]
Vyšné Jamnícke	VJ	1839	3.6	0.4	40	40	1.00	1.00	9	4.0	0.01	0.42	0.42	no	N/A
*D. galeata*															
Morskie Oko	MO	1395	50.8	34.9	36	2	0.06 (0.89)	0.06 (0.97)	6	1.7	-0.90	0.26	0.50	yes	yes [29]
Štrbské	SP	1346	20.3	19.7	38	19	0.50 (1.00)	0.91 (1.00)	9	4.3	-0.14	0.60	0.70	yes	yes [50]
Nižné Žabie Bielovodské	NZB	1675	20.5	4.7	40	2	0.05 (0.88)	0.06 (0.97)	6	1.7	-0.99	0.25	0.50	yes	yes [51]

For *D. galeata *populations, observed values of clonal richness and clonal diversity are followed in parentheses by the values obtained from datasets (N = 40) simulating random mating of genotypes detected in the analysed samples. Z_max_: maximal depth; N: number of analysed individuals; MLG: number of observed multilocus genotypes; clonal richness: MLG/N; clonal diversity: the complement of the maximum likelihood estimator of Simpson's index, (1-D); polym. loci: number of polymorphic microsatellite loci analysed; alleles/locus: polymorphism of the studied loci given as the average number of alleles detected per locus; F_IS_: fixation index; He, Ho: expected and observed heterozygosity; HWE dev.: significance of the test of deviations from Hardy-Weinberg equilibrium expectations (p < 0.05 after correction for multiple testing); N/A: data on overwintering not available. More details about conditions in the lakes, particularly on their chemistry, are given in [52].

The survival of parthenogenetic females, even at low densities, may have profound consequences for population structure, as such females have a short-term advantage at the beginning of the growing season compared to dormant eggs [5]. This advantage may be further enhanced if numerous individuals of the same clone overwinter, as can be expected after a prolonged period of clonal erosion. Using nine microsatellite loci, we assessed the late-summer clonal structure in three populations of each of the above-mentioned *Daphnia *species in order to reveal whether presumed differences in the duration of clonal reproduction (due to different overwintering strategy) are reflected in clonal richness and diversity.

## Results

**Figure 2:**
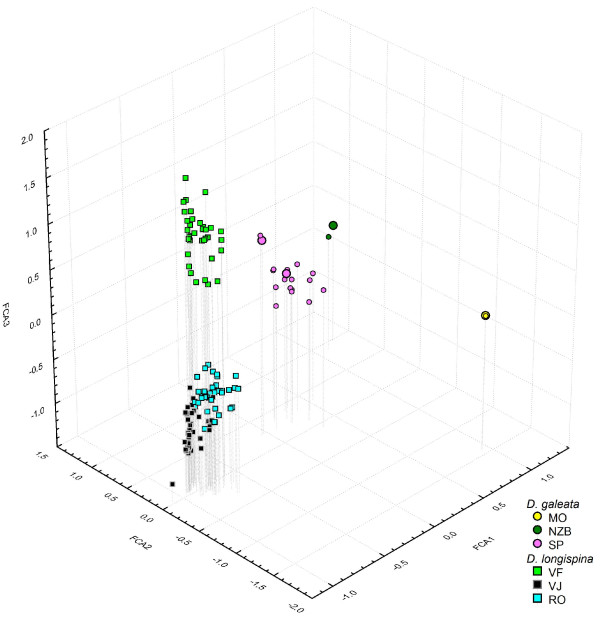
**Factorial correspondence analysis of all detected *Daphnia *multilocus genotypes, based on allelic variation at 9 microsatellite loci**. MLGs that were detected in more than 5 individuals are shown as enlarged symbols. Note that the two points representing MLGs from Morskie Oko (MO) overlap. Lake abbreviations are explained in Table 1.

Populations of both species strikingly differed in their clonal composition (Table 1, Figure 2). *D. longispina *populations showed very high clonal richness as well as diversity in the analysed samples. Clonal richness, calculated as the number of multilocus genotypes (MLGs) detected in the sample divided by the total number of studied individuals in each population (MLG/N), ranged from 0.97 to 1.00; clonal diversity, calculated as the complement of the maximum likelihood estimator of the Simpson's index, (1-D), reached the maximum possible value of 1.00 in all three populations. As these values indicate, almost every single analysed *D. longispina *individual had a different multilocus genotype (MLG), with the exception of two individuals with an identical MLG from Vyšné Furkotské Lake. None of the *D. longispina *populations significantly deviated from expectations of the Hardy-Weinberg equilibrium (HWE). The observed among-population differentiation within this species was high (D*_est _*= 0.33). These patterns are apparent in the factorial correspondence analysis (FCA) of multilocus genotypes (Figure 2), in which the three *D. longispina *populations form three mostly non-overlapping clusters composed of numerous symbols, each representing a different multilocus genotype.

*D. galeata *populations showed a very different pattern. Two populations (lakes Nižné Žabie Bielovodské and Morskie Oko) were each dominated by a single local genotype. Only one individual differed from the dominant MLG in each of these two populations (Figure 2). The resulting values of clonal richness were accordingly low (0.05 and 0.06, respectively).

The dominant MLG in these populations was heterozygous at five out of nine loci, making it highly unlikely that it harbours a substantial number of genotypes produced by sexual reproduction within the clone (i.e., due to selfing). Moreover, the percentage of polymorphic loci and allele richness in those populations was sufficient to detect possible sexually-produced MLGs. This was confirmed by the analysis of MLG richness and diversity of the randomised datasets simulating sexual reproduction within populations with the same MLG structure as recorded in the field (Table 1). Average values obtained from 25 "artificial hybrid" samples (N = 40) based on each of these two *D. galeata *populations were much higher for both clonal richness (0.88 and 1.00) and diversity (0.97-1.00) than in the original samples, approaching rather those observed in *D. longispina *populations. Furthermore, only 2.7 to 3.8% out of 1000 simulated "artificial hybrids" had MLGs identical to the respective dominant clones.

The individuals that exhibited different MLGs in these two lakes differed from the dominant ones at one locus in Nižné Žabie Bielovodské and at two loci in Morskie Oko, respectively; one allele at those variable loci was always shared with the dominant MLG. Five and four loci that were heterozygous in the dominant clones were also identical in the rare MLGs. Such differences correspond to distance classes 2 and 8, respectively, between dominant and rare MLGs, as calculated in the software GenoType. These are relatively small values; identical or lower distances between pairs of 1000 randomised MLGs were observed in only 1.8 and 5.3% of pairwise comparisons, respectively.

The third *D. galeata *population (from Štrbské Lake) was more variable than the other two, with observed values of clonal richness and diversity reaching 0.50 and 0.91, respectively. Several MLGs were detected multiple times in the sample. The two most common ones were detected seven and nine times, reaching respective proportions of 18 and 24% in the analysed sample of 38 individuals. These two locally dominant clones were heterozygous at nine and eight loci, respectively.

All three *D. galeata *populations showed significant deviations from HWE, with a strong heterozygote excess as a result of the dominance of heterozygous clones. The observed among-population differentiation expressed as the D*_est _*value was even higher than in *D. longispina *(0.42).

## Discussion

The populations of two related *Daphnia *species from the same mountain range strongly differed in their degree of clonal diversity, consistently with assumptions of their overwintering strategy. The differences in population structure between the two groups agree well with predictions of a physiologically-structured model of *Daphnia *life-history [6], which assessed the impact of clonal erosion on the genetic structure as observed by neutral markers.

Actively overwintering populations of *D. galeata*, which undergo a prolonged period of clonal selection, had much lower levels of clonal diversity, being almost completely dominated by a single clone in two cases. Moreover, the single individuals in these lakes that exhibited different multilocus genotypes differed only slightly from the dominant clones, being identical at a number of heterozygous loci. It is therefore likely that these individuals did not represent other hatchlings from sexually-produced dormant eggs but the observed differences were rather due to PCR artefacts or possibly somatic mutations of the dominant clones. This is particularly likely for Nižné Žabie Bielovodské, in which the rare MLG differed from the dominant MLG by a single allele of one locus.

In contrast with the situation in *D. galeata*, intrapopulation variation of *D. longispina *was very high, similar to what could be expected in an obligately sexual species (as demonstrated by the analysis of datasets simulating sexual reproduction in *D. galeata*; see also [4]). Although we only have data on the absence of individuals under ice for one of the three studied *D. longispina *populations, we can assume that all three populations most likely overwinter as dormant eggs, as suggested by similar genetic variation patterns. High clonal diversity suggests the limited influence of clonal erosion and the strong impact of sexual reproduction, which is consistent with the yearly re-establishment of the active population from dormant egg banks [5].

The observed patterns of clonal variation suggest that most populations we studied (apart from Štrbské Lake) represent extreme cases in a continuum of overwintering strategies. The contribution of individuals surviving the winter period to the next season's population may substantially differ, both among different localities (as observed by us) and among years within the same locality [19]. The overwintering clones may belong to the most successful ones within the population. The persistence of several genotypes during two years was observed for *Daphnia longispina *in an artificial German lake [22]; two such MLGs were the most common in both studied years (ranging from 11 to 16% of all individuals in a particular sample). We assume that this represents an intermediate situation in which both overwintering clones and new hatchlings substantially contribute to the genetic structure, a pattern probably similar to the one observed for *D. galeata *in Štrbské Lake.

A wide range of clonal diversities were observed for multiple populations of the *D. longispina *complex from various European habitats analysed by Thielsch et al. [21]. The pattern was congruent with our observations - the majority of *D. longispina *populations were characterised by very high clonal richness and diversity, but two out of three populations of *D. galeata *included in the analysis showed reduced variation. One of these was from a Dutch lake, in which we assume *Daphnia *may indeed persist year-round due to the relatively mild oceanic climate. However, low MLG diversity does not always correspond to long-term or strong clonal erosion. For example, the lowest diversity among *D. longispina *populations was reported from a small Tatra Mountain lake, Vyšné Satanie [21]. In that case, however, it was most likely due to low allelic richness and low heterozygosity (and thus limited ability to differentiate between different MLGs), as it is unlikely that harsh winter conditions in this lake allow overwintering of cladocerans in active stages. The low allele richness in that particular population more probably results from an introduction bottleneck when the population became re-established after a period of severe anthropogenic acidification [21,24].

Although we studied two different species, the overwintering strategy in the *D. longispina *complex is not necessarily species-specific. Whether or not *Daphnia *successfully survive the winter period is rather influenced by the ability to adapt to local conditions in a particular lake [18,25,26]. For example, populations of the same two species co-occurring in Lake Constance showed the opposite pattern to that observed in the Tatra Mountains: *D. longispina *(labelled *D. hyalina *in previous studies) overwintered in Lake Constance and did not invest in sexual reproduction, while *D. galeata *produced dormant eggs and disappeared from the water column in winter [18]. This was also reflected in MLG variation (based on two allozyme loci) being lower in overwintering *D. longispina *than in *D. galeata *[18].

The fact that two coexisting *Daphnia *taxa in Lake Constance differed in their overwintering strategy within a single lake suggests that this life history trait has a genetic component. Although the conspecific Tatra Mountain populations analysed here showed a similar genetic structure, this is not necessarily a consequence of genetic relatedness. The patterns of mitochondrial variation suggest that each of these species colonised the Tatra Mountains multiple times, and in only two of the studied lakes from the West Tatras, Dolné Roháčské and Vyšné Jamnícke, did they likely originate from the same source [24,27]. This pattern is well reflected in the Factorial Correspondence Analysis plot (Figure 2), in which individuals from these lakes are most similar. Furthermore, the overwintering strategy of *D. galeata *tends to be similar despite the fact that some important selection factors, such as predation pressure, strongly differ among the lakes. In two of them (Morskie Oko and Štrbské Lake), fish predation on *Daphnia *is high [24,29], while there are no fish in the third studied lake (Nižné Žabie Bielovodské).

The strategy of daphnids in the Tatra Mountain lakes is most likely directly influenced by the size and depth of lakes. In this mountain range, *D. galeata *inhabits several relatively large and deep lakes, while *D. longispina *is found in smaller ones [24]. In deeper lakes, the likelihood of successful overwintering of *Daphnia *individuals is higher, as they may survive in the deep refuge [25]. In addition, investing into dormant eggs is much less efficient, as ephippia that sink to deep parts of the lakes are much less likely to hatch [28]. The studied *D. galeata *populations from the Tatra Mountains apparently regularly overwinter, as observed in previous studies (Table 1). In fact, investment in sexual reproduction and ephippia production seems to be absent or extremely low in the deep lake Morskie Oko, where no ephippia-bearing females or males were observed during an intensive one-year study of this population [29].

Apparently, prolonged clonal reproduction is highly advantageous for *D. galeata *populations in the studied lakes. Gliwicz et al. [29] even suggested that Morskie Oko *Daphnia *are obligately asexual. Indeed, a higher frequency of obligate parthenogenetic *Daphnia *has been observed at high altitudes [e.g., [2,30]], and populations identified as *D. pulicaria *inhabiting alpine lakes in the Tatra Mountains are strictly asexual [31]. Interestingly, even asexual lineages coexisting in a single lake may differ in overwintering strategy [29]. However, we consider it unlikely that *D. galeata *populations in the Tatra Mountains are obligate parthenogens, in the sense that they produce dormant eggs parthenogenetically (see [2] for an overview). To our knowledge, this reproduction mode has never been convincingly shown in the *D. longispina *complex, and it is unlikely that it would have arisen at least twice independently in the Tatra Mountain lakes that were likely colonized by *D. galeata *from different sources [24,27]. It seems rather that local *D. galeata *populations remain in the parthenogenetic life cycle phase only, and have stopped investing in sexual reproduction altogether.

Moreover, we observed reduced levels of clonal diversity in the *D. galeata *population from Štrbské Lake, a locality in which the *Daphnia *history in recent decades is relatively well known. Štrbské Lake was colonized by *D. galeata *in the second half of the 20^th ^century, and subsequently gradually replaced the resident population of *D. longispina *[24,32]. From this locality, we have direct evidence for the production of ephippia in *D. galeata*, as well-preserved dormant eggs of this species extracted from various sediment layers from this lake were analysed genetically [32]. Interestingly, *D. longispina *also could be found in Štrbské Lake in winter at the beginning of the 20^th ^century [33], which suggests generally favourable conditions for overwintering at this locality. Historical data also confirm that *D. galeata *was already present in Morskie Oko a century ago [24,33]. It is possible that the higher clonal diversity observed in *D. galeata *from Štrbské Lake is due to the much younger age of this population, as a result of which clonal selection has not yet eroded genetic diversity to the same extent as in the two other populations.

Alternatively, this population may have a generally higher tendency to recruit from dormant eggs than the other two studied *D. galeata *populations, either due to local environmental factors or due to its genetic background. In particular, it is possible that the local population was influenced by introgression from *D. longispina *during the period of species replacement (see [34]), as also suggested by the intermediate position of some MLGs from Štrbské Lake in the Factorial Correspondence Analysis (Figure 2). However, we presume that environmental conditions are more likely driving the *Daphnia *overwintering strategy than the taxonomic composition of the populations.

Apart from reductions of clonal diversity, another predicted consequence of prolonged clonal erosion is deviation from the Hardy-Weinberg equilibrium. Simulation data [6] suggest that the typical result of clonal selection is a heterozygote excess even in the absence of any selection advantage for heterozygotes. Indeed, we observed strong heterozygote excess in all three studied *D. galeata *populations that survive winter as active animals, in contrast to *D. longispina *populations. The third consequence of clonal erosion may be among-population differentiation [6]. This was high for both studied species, but *D. galeata *did show higher differentiation (D*_est _*= 0.42, in comparison with 0.33 for *D. longispina*), which would conform to the prediction. In this case, however, factors other than clonal selection are probably more important, in particular persistent founder effects [4,35]. Patterns of mitochondrial DNA variation suggest multiple independent colonisations of Tatra Mountain lakes within each species [24,27], and the high among-population differentiation is likely a direct consequence.

Our data show that the genetic diversity of a *Daphnia *population may be strongly influenced by the choice of reproductive strategy during unfavourable periods, and can show greatly different patterns among populations living in close proximity to one another, depending on local environmental conditions. What are the benefits and costs of an active overwintering strategy? Females surviving under the ice are ready to start reproduction as soon as conditions improve in spring, which is a great advantage against genotypes hatching from dormant eggs [26]. If seasonal changes in a particular lake are sufficiently predictable over a long period, overwintering genotypes may eventually prevail in the population if no selection force acts in a negative frequency-dependent manner (e.g., microparasites; see [36]). Furthermore, immigrating genotypes less adapted to local conditions would be highly unlikely to establish [37], and even if so, they would not succeed in successful interbreeding with the locally adapted clones.

In extreme cases, long-term clonal erosion may result in the overwhelming dominance of a single clone, as observed in two of the three studied *D. galeata *populations. However, if these clones invest little or nothing into the production of dormant eggs, this may be an evolutionary trap. Ephippia produced earlier in the population are effectively buried in the sediment, and re-establishment from the dormant egg bank may be difficult (but see [38]). A low effective population size may then prevent microevolutionary changes necessary for adaptation to new selective forces, such as the introduction of new predators or parasites.

## Conclusions

Our study demonstrates how different life history adaptations to unfavourable periods may impact the population genetic diversity in cyclical parthenogens. Two closely related *Daphnia *species living in mountain lakes differed in overwintering strategy, which influences the length of the period of clonal erosion and frequency of sexual reproduction. Consequently, strikingly different patterns of genetic diversity were observed in populations of the two species. The use of microsatellite markers, which are variable enough to reveal true clonal structures in cyclical parthenogen populations, revealed that populations surviving unfavourable conditions as active individuals may be dominated by a single clone. Such an extreme reduction of clonal diversity due to long-term clonal erosion may have profound impacts on the microevolutionary potential of such populations.

## Methods

There are over 250 glacier lakes in the Tatra Mountains, but only a minority of these are inhabited by *Daphnia *populations. The two studied species are locally the commonest members of the *D. longispina *complex (more details on zooplankton of the Tatra Mountain lakes are given in [24,39]). We studied six lakes, three inhabited by *D. longispina*, and three by *D. galeata *(Figure 1).

During winter zooplankton sampling of Vyšné Furkotské Lake, inhabited by *D. longispina*, no individuals were found [40]; likely, this population survives winter as dormant eggs encapsulated in protective ephippia in the lake sediment. Winter zooplankton was never studied in the other two *D. longispina *lakes, Vyšné Jamnické and Dolné Roháčské (E. Stuchlík, pers. comm.), but given their similarity to Vyšné Furkotské Lake (Table 1), it is likely that their *Daphnia *populations are under similar selection pressures regarding overwintering strategy. In contrast, all three *D. galeata *populations, which were sampled from larger and deeper lakes with slightly lower elevation than *D. longispina*, have been documented to overwinter as adult females under the ice cover (see references in Table 1); in at least one case, they were able to reproduce in such conditions [29].

All studied lakes were sampled in September 2007, close to the end of the main growing season (which is delayed and much shorter in these alpine environments in comparison with lowland habitats). Sampling during this period ensured that clonal structure that might have arisen by hatching early in the growing season (which lasts, depending on altitude, between approx. May/June to October) would be noticed genetically. Samples were collected by tows of a plankton net from the lake shore or from a rubber boat by vertical hauls from the depth, and preserved in 96% ethanol. DNA from individuals was extracted in 100 μl proteinase K solution, using the protocol from [41].

Genotypes of individuals were determined by analysis of nine microsatellite loci described previously [42], amplified in a single multiplex reaction. PCRs of the 11 μl volume consisted of 10 μl of master-mix (2.9 μl of primer mix: 0.4 μM Swid15, 0,3 μM Swid1, 0.05 μM Dp281NB, 0.2 μM Dp196NB, 0.3 μM Swid12, 0.3 μM Dp512, 0.2 μM Swid10, 0.3 μM Swid14, 0.3 μM Dgm109; 5.5 μl of MP-mix (Quiagen Multiplex PCR Kit); 1.6 μl of water) and 1 μl of DNA extract. The PCR cycle consisted of the following steps: initial denaturation at 95°C for 15 min, 30 cycles of denaturation at 94°C for 0.5 min, annealing at 54°C for 1.5 min and elongation at 72°C for 1 min, with final elongation at 60°C for 30 min. Fragment analysis of PCR products was performed on the capillary sequencer ABI 3130 with the Gene Scan Liz 500 size standard, and individuals were subsequently genotyped in GeneMapper 4.0. (Applied Biosystems, Foster City, CA). Individuals differing from dominant MLGs in *D. galeata *populations were carefully checked to rule out scoring errors.

Each individual was characterized by its multilocus genotype (MLG), and the patterns of MLG similarity were visualised by factorial correspondence analysis (FCA) calculated in Genetix v4.03 [43]. Clonal richness was expressed as the number of MLGs detected in the sample divided by the total number of studied individuals in each population (MLG/N), the clonal diversity as a complement of the maximum likelihood estimator of Simpson's index (1-D), calculated in the program SPADE [44].

To compare the observed clonal structures in each *D. galeata *population with those expected under the strong influence of recruitment from dormant eggs, we used the program HYBRIDLAB [45] to generate a randomised dataset simulating the result of sexual reproduction of analysed individuals, i.e., multilocus genotypes of artificial hybrids (assuming independent segregation of studied microsatellite loci). We then calculated the above-mentioned measures of clonal richness and diversity for 25 sets of 40 "artificial hybrids" from each *D. galeata *population, and compared the averaged values with those from field samples of both *Daphnia *species.

Additionally, we evaluated whether rare genotypes observed in populations dominated by a single MLG more likely arose independently, or resulted from a somatic mutation or methodological artefact. The software GenoType [46] was used to calculate a pairwise distance matrix between individual multilocus genotypes within each sample, assuming a stepwise mutation model. The distribution of such pairwise distances may be used to select a threshold that defines the maximum difference between two MLGs at which they are still assigned to the same clonal lineage [47], i.e., to neglect differences caused by PCR artefacts, scoring errors, or somatic mutations. We compared the distance class distribution of simulated datasets of 1000 "artificial hybrids" (generated as above) with that observed in *D. galeata *populations dominated by a single clone.

The extent of deviations from the Hardy-Weinberg equilibrium, which are predicted under the scenario of strong clonal erosion [6], were tested in the software Hwclon [7] using Monte Carlo simulations (20 batches of 500 permutations). As there were six repeated tests, we applied sequential Bonferroni correction when assessing the significance of the results. Genetic differentiation among populations, based on the evaluated microsatellite markers, was assessed by means of D*_est _*[48], using the SMOGD software [49].

## Authors' contributions

AP and EH designed the study and did the sampling. EH and JM carried out the molecular work. All authors contributed to data analyses and preparation of the manuscript, read and approved the final version.

## References

[B1] De Meester L Gómez A Simon JC Moya A, Font E Evolutionary and ecological genetics of cyclical parthenogens Evolution from Molecules to Ecosystems 2004 Oxford University Press 122 134

[B2] Decaestecker E De Meester L Mergeay J Schön I, Martens K, van Dijk P Cyclical parthenogenesis in *Daphnia*: sexual versus asexual reproduction Lost Sex: The Evolutionary Biology of Parthenogenesis 2009 Springer 295 316

[B3] Gyllström M Hansson LA Dormancy in freshwater zooplankton: Induction, termination and the importance of benthic-pelagic coupling Aquat Sci 2004 66 274 295

[B4] De Meester L Local genetic differentiation and adaptation in freshwater zooplankton populations: patterns and processes Ecoscience 1996 3 385 399

[B5] De Meester L Vanoverbeke J De Gelas K Ortells R Spaak P Genetic structure of cyclic parthenogenetic zooplankton populations - a conceptual framework Arch Hydrobiol 2006 167 217 244 10.1127/0003-9136/2006/0167-0217

[B6] Vanoverbeke J De Meester L Clonal erosion and genetic drift in cyclical parthenogens - the interplay between neutral and selective processes J Evol Biol 2010 23 997 1012 10.1111/j.1420-9101.2010.01970.x 20345816

[B7] De Meester L Vanoverbeke J An uncoupling of male and sexual egg production leads to reduced inbreeding in the cyclical parthenogen *Daphnia* Proc R Soc Lond B 1999 266 2471 2477 10.1098/rspb.1999.0948 PMC1690476 10693817

[B8] Vanoverbeke J De Meester L Among-populational genetic differentiation in the cyclical parthenogen *Daphnia magna *(Crustacea, Anomopoda) and its relation to geographic distance and clonal diversity Hydrobiologia 1997 360 135 142 10.1023/A:1003160903708

[B9] Brendonck L De Meester L Egg banks in freshwater zooplankton: evolutionary and ecological archives in the sediment Hydrobiologia 2003 491 65 84

[B10] Cáceres CE Hartway C Paczlot KA Inbreeding depression varies with investment in sex in a facultative parthenogen Evolution 2009 63 2474 2480 10.1111/j.1558-5646.2009.00707.x 19473400

[B11] Gómez A Carvalho GR Sex, parthenogenesis and the genetic structure of rotifers: microsatellite analysis of contemporary and resting egg bank population Mol Ecol 2000 9 203 214 10.1046/j.1365-294x.2000.00849.x 10672164

[B12] Sunnucks P DeBarro PJ Lushai G Maclean N Hales D Genetic structure of an aphid studied using microsattelites: Cyclic parthenogenesis, differentiated lineages and host speciation Mol Ecol 1997 6 1059 1073 10.1046/j.1365-294X.1997.00280.x 9394464

[B13] Hartnett DC Bazzaz FA The genet and ramet population dynamics of *Solidago canadensis *in an abandoned field J Ecol 1985 73 407 413 10.2307/2260483

[B14] Janko K Drozd P Flegr J Pannell JR Clonal turnover versus clonal decay: a null model for observed patterns of asexual longevity, diversity and distribution Evolution 2008 62 1264 1270 10.1111/j.1558-5646.2008.00359.x 18315576

[B15] Janko K Drozd P Eisner J Do clones degenerate over time? Explaining the genetic variability of asexuals through population genetic models Biology Direct 2011 6 17 10.1186/1745-6150-6-17 21371316 PMC3064643

[B16] Lampert W Lampert KP Larsson P Coexisting overwintering strategies in *Daphnia pulex*: A test of genetic differences and growth responses Limnol Oceanogr 2010 55 1893 1900 10.4319/lo.2010.55.5.1893

[B17] Zaffagnini F Peters RH, de Bernardi R Reproduction in *Daphnia* Daphnia 1987 45 245 284 Mem Ist Ital Idrobiol

[B18] Jankowski T Straile D Allochronic differentiation among *Daphnia *species, hybrids and backcrosses: the importance of sexual reproduction for population dynamics and genetic architecture J Evol Biol 2004 17 312 321 15009265

[B19] Zeis B Horn W Gigengack U Koch M Paul RJ A major shift in *Daphnia *genetic structure after the first ice-free winter in a German reservoir Freshw Biol 2010 55 2296 2304

[B20] Spaak P Temporal changes in the genetic structure of the *Daphnia *species complex in Tjeukemeer, with evidence for backcrossing Heredity 1996 76 539 548 10.1038/hdy.1996.77

[B21] Thielsch A Brede N Petrusek A De Meester L Schwenk K Contribution of cyclic parthenogenesis and colonization history to population structure in *Daphnia* Mol Ecol 2009 18 1616 1628 10.1111/j.1365-294X.2009.04130.x 19298264

[B22] Yin M Wolinska J Giessler S Clonal diversity, clonal persistence and rapid taxon replacement in natural populations of species and hybrids of the *Daphnia longispina *complex Mol Ecol 2010 19 4168 4178 10.1111/j.1365-294X.2010.04807.x 20819161

[B23] Petrusek A Hobæk A Nilssen JP Skage M Černý M Brede N Schwenk K A taxonomic reappraisal of the European *Daphnia longispina *complex (Crustacea, Cladocera, Anomopoda) Zool Scr 2008 37 507 519 10.1111/j.1463-6409.2008.00336.x

[B24] Petrusek A Černý M Mergeay J Schwenk K *Daphnia *in the Tatra Mountain lakes: multiple colonisation and hidden species diversity revealed by molecular markers Fundam Appl Limnol 2007 169 279 291 10.1127/1863-9135/2007/0169-0279

[B25] de Senertpont Domis LN Mooij WM Hülsmann S van Nes EH Scheffer M Can overwintering versus diapausing strategy in *Daphnia *determine match-mismatch events in zooplankton-algae interactions? Oecologia 2007 150 682 698 17024385 10.1007/s00442-006-0549-2

[B26] Rellstab C Spaak P Lake origin determines *Daphnia *population growth under winter conditions J Plankton Res 2009 31 261 271

[B27] Hamrová E Genetic structure of the *Daphnia longispina *complex in European mountain lakes Ph.D. thesis 2011 Department of Ecology, Faculty of Science, Charles University in Prague

[B28] Cáceres CE Tessier AJ To sink or swim: Variable diapause strategies among *Daphnia *species Limnol Oceanogr 2004 49 1333 1340 10.4319/lo.2004.49.4_part_2.1333

[B29] Gliwicz ZM Slusarczyk A Slusarczyk M Life history synchronization in a long-lifespan single-cohort *Daphnia *population in a fishless alpine lake Oecologia 2001 128 368 378 10.1007/s004420100673 24549906

[B30] Aguilera X Mergeay J Wollebrants A Declerck S De Meester L Asexuality and polyploidy in *Daphnia *from the tropical Andes Limnol Oceanogr 2007 52 2079 2088 10.4319/lo.2007.52.5.2079

[B31] Dufresne F Marková S Vergilino V Ventura M Kotlík P Diversity in the reproductive modes of European *Daphnia pulicaria *deviates from the geographical parthenogenesis PLoS ONE 2011 6 e20049 10.1371/journal.pone.0020049 21655327 PMC3104988

[B32] Hamrová E Goliáš V Petrusek A Identifying century-old long-spined *Daphnia*: species replacement in a mountain lake characterised by paleogenetic methods Hydrobiologia 2010 643 97 106 10.1007/s10750-010-0127-9

[B33] Lityński A Revision der Cladocerenfauna der Tatra-Seen. I. Teil. Daphnidae Bull int Acad Sci Cracovie, Cl Sci Math Nat, ser B 1913 1913 566 623

[B34] Brede N Sandrock C Straile D Spaak P Jankowski T Streit B Schwenk K The impact of human-made ecological changes on the genetic architecture of *Daphnia *species Proc Natl Acad Sci USA 2009 106 4758 4763 10.1073/pnas.0807187106 19273852 PMC2653563

[B35] Boileau MG Hebert PDN Schwartz SS Non-equilibrium gene frequency divergence: persistent founder effect in natural populations J Evol Biol 1992 5 25 39 10.1046/j.1420-9101.1992.5010025.x

[B36] Wolinska J Spaak P The cost of being common: evidence from natural *Daphnia *populations Evolution 2009 63 1893 1901 10.1111/j.1558-5646.2009.00663.x 19228186

[B37] De Meester L Gómez A Okamura B Schwenk K The Monopolization Hypothesis and the dispersal-gene flow paradox in aquatic organisms Acta Oecol 2002 23 121 135 10.1016/S1146-609X(02)01145-1

[B38] Mergeay J Vanoverbeke J Verschuren D De Meester L Extinction, recolonisation and dispersal through time in a planktonic crustacean Ecology 2007 88 3032 3043 10.1890/06-1538.1 18229838

[B39] Hořická Z Stuchlík E Hudec I Černý M Fott J Acidification and the structure of crustacean zooplankton in mountain lakes: The Tatra Mountains (Slovakia, Poland) Biologia 2006 61 S121 S134 10.2478/s11756-006-0125-6

[B40] Blažka P Některé fysiologické charakteristiky tatranských korýšů [Some physiological characteristics of the Tatra crustaceans] Zbor prác o Tatr Nár parku 1964 7 227 231

[B41] Schwenk K Sand A Boersma M Brehm M Mader E Offerhaus D Spaak P Genetic markers, genealogies and biogeographic patterns in the Cladocera Aquat Ecol 1998 32 37 51 10.1023/A:1009939901198

[B42] Brede N Thielsch A Sandrock C Spaak P Keller B Streit B Schwenk K Microsatellite markers for European *Daphnia* Mol Ecol Notes 2006 6 536 539 10.1111/j.1471-8286.2005.01218.x

[B43] Belkhir K Borsa P Chikhi L Raufaste N Bonhomme F Genetix 4.05, logiciel sous Windows TM pour la génétique des populations 1996 Laboratoire Génome, Populations, Interactions, CNRS UMR 5000, Université de Montpellier II, Montpellier, France http://www.genetix.univ-montp2.fr

[B44] Chao A Shen T-J Program SPADE (Species Prediction And Diversity Estimation) 2003 http://chao.stat.nthu.edu.tw

[B45] Nielsen EE Bach LA Kotlicki P HYBRIDLAB (version 1.0): a program for generating simulated hybrids from population samples Mol Ecol Notes 2006 6 971 973 10.1111/j.1471-8286.2006.01433.x

[B46] Meirmans PG Van Tienderen PH GenoType and GenoDive: two programs for the analysis of genetic diversity of asexual organisms Mol Ecol Notes 2004 4 792 794 10.1111/j.1471-8286.2004.00770.x

[B47] Rogstad SH Keane B Beresh J Genetic variation across VNTR loci in central North American *Taraxacum *surveyed at different spatial scales Plant Ecol 2002 161 111 121 10.1023/A:1020301011283

[B48] Jost L G_ST _and its relatives do not measure differentiation Mol Ecol 2008 17 4015 4026 10.1111/j.1365-294X.2008.03887.x 19238703

[B49] Crawford NG SMOGD: Software for the measurement of genetic diversity Mol Ecol Res 2010 10 556 557 10.1111/j.1755-0998.2009.02801.x 21565057

[B50] Ertl M Príspevok k poznaniu zimného zooplanktonu Štrbského plesa [Contribution to the knowledge of winter zooplankton of Štrbské pleso lake] Biologia 1963 18 787 791

[B51] Kneslová P Dargocká J Stuchlík E Zooplankton osmi různě acidifikovaných ples ve Vysokých Tatrách [Zooplankton of eight High Tatra Mountain lakes in different stage of acidification] Štúd o Tatr nár parku 1997 2 123 134

[B52] Kopáček J Stuchlík E Hardekopf D Chemical composition of the Tatra Mountain lakes: Recovery from acidification Biologia 2006 61 S21 S33 10.2478/s11756-006-0117-6

